# Society Meetings

**Published:** 1916-08

**Authors:** 


### SOCIETY MEETINGS

**Brief reports and announcements of meetings of societies of Western New York, and those of general scope, are requested from Secretaries. Copy should be on hand the fifteenth of the month. Full transactions will be published at cost of composition.**

---

**DOCTOR: Is your Society properly represented here? If not, please bring our standing announcement to the attention of the members.**

---

**Lake Keuka Medical & Surgical Assn.** 17th annual meeting at Keuka College Auditorium, July 13-14. Program:

1. President's Address, Homer J. Knickerbocker, M. D., F. A. C. S., Geneva, N. Y.
2. "The Control of Typhoid Fever in the State," Fred M. Meader, M. D., Albany, N. Y.
3. "Manner of Infection in Some Cases of Puerpural Sepsis," Charles F. Neider, M. D., Geneva, N. Y.

4. ''Concealed Infections of the Genito-Urinary Tract,'' Arthur H. Paine, M. D., Rochester, N. Y.

Discussion opened by Thos F. Laurie, M. D., Auburn, N. Y.

5. ''Prognostic Value and Clinical Application of Studies in Retention,'' Richard N. DeNiord, M. D., Buffalo, N. Y.

Discussion opened by Frederick J. Parmenter, M. D., F. A. C. S., Buffalo, N. Y.

6. ''The Patient Before, During and After the Anesthetic,'' Charles S. Hunt, M. D., New York.

7. ''The Value of the Clinical History in the Diagnosis of Gastric and Duodenal Ulcer,'' George Eusterman, M. D., Rochester, Minn.

8. ''Roentgenological Findings in the Diagnosis of Intraabdominal Conditions,'' I. Harris Levy, M. D., Syracuse, N. Y.

9. ''Methods and Results in Surgery of the Stomach and Intestines,'' George W. Crile, M. D., F. A. C. S., Cleveland, O.

Discussion of papers of Drs. Eusterman, Levy and Crile will be opened by Donald Guthrie, M. D., F. A. C. S., Sayre, Pa.; John M. Garratt, M. D., Buffalo, N. Y., and Ross G. Loop, M. D., F. A. C. S., Elmira, N. Y

10 ''The Nature of Acidosis in General, with Report of Observations on Acidosis in Diabetes,'' Donald D. VanSlyke, Ph. D., New York.

Discussion opened by John R. Williams, M. D., Rochester, N. Y.

11. ''Pathological Diagnosis,'' Burton T. Simpson, M. D., Buffalo, N. Y. Read by title.

12. ''Spinal Injuries, Commonly Known as Broken Neck or Back. A Preliminary Report Based on Fifty-two Cases,'' Lee A. Whitney, M. D., Rochester, N. Y.

13. ''Acute Mastoiditis. Diagnosis and Treatment, with Special Reference to X-ray Diagnosis,'' John F. Fairbairn, M. D., F. A. C. S., Buffalo, N. Y.

14. ''Gynecologic Diagnosis,'' Aaron B. Miller, M. D., F. A. C. S., Syracuse, N. Y. Read by title.

Officers for 1916: Pres. H. J. Knickerbocker, Geneva; V. P. J. R. Wiseman, Syracuse; Sec.-Treas. E. C. Foster, Penn Yan; Rec. Sec. W. W. Smith, Avoca; Committee of Arrangements, E. C. Foster, Penn Yan; P. L. Alden, Hammondsport; Fred Maloney, Dundee. Dr. I. Harris Levy of Syracuse was elected President and L. C. Lewis of Belmont V. P. for 1917, the secretaries being re-elected. Dr. Lesser Kauffmann of Buffalo was elected a member of the Council. Cayuga, Cortland and Tompkins Cos. were added to the list, making a total of 20.

---

**The Rochester Academy of Medicine** held a special memorial meeting in honor of the late Dr. John F. W. Whitbeck, July 5.

---

**The Cremation Association of America** will hold its 4th annual convention at the Hotel Gibson, Cincinnati, Aug. 24 and 25. The number of crematories in Germany is now 48. The U. S. has 53 with 2 in contemplation. German incinerations totaled 76,350 through 1915, American 86,006 through 1913. The first crematory in the U. S. was built by Dr. Francis Julius Le Moyne, a graduate of the University of Pennsylvania, at his own expense. All interested are invited to attend and may secure associate membership by remitting $1 to E. P. Samson, 433 Sixth Ave., Pittsburgh. The Secretary is Dr. Hugo Erichsen, 240 Chandler Ave., Detroit.

---

**The Medical Societies of the Counties of Allegany, Genesee, Livingston and Wyoming** held a joint meeting at Glen Iris, Letchworth Park, July 25. The program was as follows:

"Methods of Resuscitation; with a Demonstration of the Pharyngeal Insufflation Apparatus for Artificial Respiration in Man," by Dr. S. J. Meltzer, Director Rockefeller Institute for Medical Research, New York City.

"Modern Methods of Teaching Surgery." (Motion Pictures). Bloodless Amputation of the Hip Joint, by Dr. John A. Bodine. Nephrotomy and Nephrectomy, by Dr. Charles H. Chetwood. Radical Cure of Inguinal Hernia, by Dr. John A. Bodine, New York, Polyclinic Medical School, New York City.

---

The transactions of the **Alumni Ass'n of the Medical Dept., University of Buffalo,** promised for this issue, have been delayed, as some of those responsible for details of the minutes are out of town, on military duty or private business. Space was held till July 24, when it was necessary to close the forms. The transactions will probably be published in the Sept. issue.

